# Assessing and Improving the Reproducibility of Cerebrovascular Reactivity Evaluations in Healthy Subjects Using Short-Breath-Hold fMRI

**DOI:** 10.3390/diagnostics15151946

**Published:** 2025-08-03

**Authors:** Emely Renger, Till-Karsten Hauser, Uwe Klose, Ulrike Ernemann, Leonie Zerweck

**Affiliations:** Department of Diagnostic and Interventional Neuroradiology, University Hospital Tuebingen, 72076 Tuebingen, Germany; till-karsten.hauser@med.uni-tuebingen.de (T.-K.H.); uwe.klose@med.uni-tuebingen.de (U.K.); ulrike.ernemann@med.uni-tuebingen.de (U.E.); leonie.zerweck@med.uni-tuebingen.de (L.Z.)

**Keywords:** cerebrovascular reactivity, breath-hold fMRI, reproducibility, short breath-hold periods

## Abstract

**Background/Objectives**: Cerebrovascular reactivity (CVR) is a key marker of cerebrovascular function, facilitating the early detection of neurovascular dysfunction. Breath-hold functional MRI (bh-fMRI) is a non-invasive method for assessing CVR. This study evaluates the reproducibility of bh-fMRI using short breath-hold periods, which are practical for clinical use. **Methods**: In a prospective study, 50 healthy subjects underwent three self-paced, end-expiration bh-fMRI sessions with 9 s breath-hold periods at 3T. A 30 min break between the second and third sessions was included. The reproducibility of the percentage signal change (PSC) in predefined volumes of interest for a ±0 s, ±3 s and ±6 s interval around the cerebellar peak (IAP)) was evaluated. The intraclass correlation coefficient (ICC) and the intra-personal coefficient of variation (CV_intra_) were calculated between the individual sessions. **Results**: This study demonstrated excellent reproducibility, with an ICC (2, k) for a ±0 s IAP across all sessions at 0.887 (95% CI: 0.882–0.892). The ICC values remained within an excellent range even when the participants left the scanner between sessions. The CV_intra_ for the ±0 s IAP (14.54% ± 8.54%) remained below the 33% fiducial limit. A larger IAP revealed higher ICC values but higher CV_intra_ values and lower PSC values. **Conclusions**: Bh-fMRI with 9 s breath-hold periods yields highly reproducible CVR assessments, supporting its feasibility for clinical implementation.

## 1. Introduction

Cerebrovascular reactivity (CVR) is a pivotal physiological parameter that demonstrates the ability of the cerebral vessels to adjust the cerebral blood flow (CBF) in response to physiological stimuli, pathological vascular alterations such as stenoses, and vasoactive agents [1,2,3]. This adaptability is crucial for maintaining CBF and supporting neuronal activity under dynamic metabolic and physiological demands [2].

Consequently, CVR has emerged as a surrogate marker of vascular function and is indicative of enhanced vascular wellbeing [4,5]. It is utilized with increasing frequency to evaluate cerebrovascular health and detect early indications of dysfunction in conditions such as Moyamoya Angiopathy (MMA) and stroke [6,7], proximal arterial stenosis [8,9], intracranial artery stenosis [10,11], hypertension [12], and traumatic brain injury [13,14].

Whilst a variety of imaging techniques can be utilized to measure CVR [15,16,17,18], blood-oxygenation-level-dependent functional magnetic resonance imaging (BOLD fMRI) with hypercapnic stimulation offers a number of advantages due to its status as a non-invasive method without radiation exposure [1,3,5,19]. In comparison with the diagnostic gold standard, [^15^O]water positron emission tomography (PET), fMRI has been shown to be a more cost-effective option, as well as being a procedure that is more widely available [1,19,20].

In healthy brain parenchyma, hypercapnia induces a shift in the concentration of CO_2_, leading to acidosis [21] in the interstitial space. This causes vasodilatation with increased cerebral perfusion [22], while the cerebral metabolic rate of oxygen (CMRO_2_) remains relatively unchanged [21]. The increase in blood flow and unchanged oxygen consumption lead to a decrease in the concentration of paramagnetic deoxyhemoglobin in the venules [23], causing a change in magnetic susceptibility, which in turn leads to an increase in the BOLD signal [21,24,25]. Regions with a high cerebral perfusion reserve show an increased BOLD signal, while areas with diminished CVR show a minimal rise or no rise in blood flow. In such cases, it is hypothesized that the cerebral vessels are already maximally dilated, resulting in exhaustion of the cerebrovascular reserve capacity and the inability of the CBF to increase further [1]. In certain instances, a phenomenon referred to as the steal effect can be observed. This occurs when healthy regions of the brain receive an increased blood supply, resulting in a decreased blood flow to regions that show severely impaired CVR. These regions therefore show negative CVR values [26].

Hypercapnia fMRI studies often rely on controlled CO_2_ inhalation to induce vascular responses [1]. Breath-holding-induced hypercapnia is a robust and practical stimulus that can be used to evaluate CVR without the need for external gas administration or patient monitoring, which can be uncomfortable for patients [27]. The breath-hold fMRI (bh-fMRI) method has the benefit of being simple to implement and non-reliant on specialized equipment, making it accessible in a broad range of clinical and research environments [28]. Moreover bh-fMRI has shown similar results compared to those for fMRI with CO_2_ inhalation [29,30] and the diagnostic gold standard, [^15^O]water PET [11,31].

However, despite the increasing application of bh-fMRI, its reproducibility for CVR measurements has not been extensively evaluated. Reproducibility is a fundamental requirement for the establishment of any imaging protocol as a reliable biomarker. In the context of fMRI-based CVR measurements, reproducibility ensures that the vascular responses observed are consistent across repeated sessions and not confounded by experimental variability.

The primary objective of this study is to evaluate the within-day reproducibility of bh-fMRI CVR assessments in a healthy cohort. We distinguish our approach from others by using short breath-hold periods (bh periods) of 9 s at end-expiration, shorter than those in other studies, which have normally used longer periods [32,33,34]. These longer bh periods can be more demanding and less tolerable for patients [35], and previous studies have shown that above 9 s, there is no additional increase in the resulting BOLD signal [20]. Moreover, we included a break, in which the participants had to leave the MR scanner, so that we could also evaluate the effect of patient repositioning and the examination time on the calculated CVR maps.

## 2. Materials and Methods

### 2.1. The Participants

A prospective fMRI study was conducted in a group of healthy subjects. Participants between 18 and 80 years who provided informed consent were included. The exclusion criteria encompassed MRI contraindications (e.g., metal implants, pacemakers), neurological or psychiatric disorders, conditions preventing 30 min of lying still, the inability to follow instructions (e.g., cognitive or cardiopulmonary limitations), and pregnancy. The study protocol was approved by the local ethics committee.

### 2.2. MRI Data Acquisition

Imaging was conducted in a 3 T MR scanner (Magnetom Prisma, Siemens, Erlangen, Germany) utilizing a standard 20-channel head coil. Subjects were positioned in a head-first supine position on the scanner table. T2*-weighted echo-planar sequences with the following parameters were used: TR = 3000 ms; TE = 36 ms; flip angle = 90°; matrix size = 96 × 96; 3 mm slice thickness; 34 slices in interleaved ascending order; FOV = 245 mm; resolution = 2.6 × 2.6 × 3.0 mm^3^; TA = 9:14 min; and 181 measurements.

**Figure 1:**
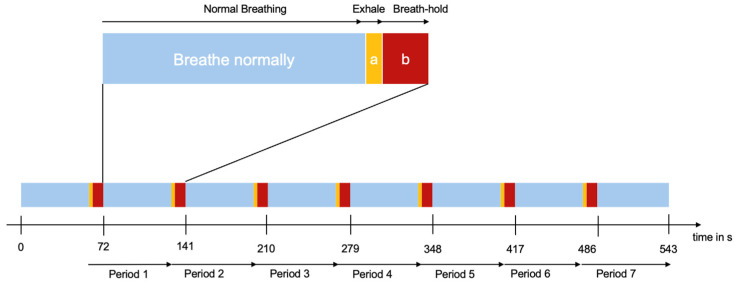
A schematic representation of the breath-hold paradigm: 60 s of normal breathing was followed by seven cycles, each consisting of a phase of normal breathing (57 s), followed by 3 s of exhalation (a) and 9 s of breath-holding (b).

The bh-fMRI protocol commenced with 60 s of regular self-paced breathing followed by seven repetitive cycles of 3 s of expiration, 9 s of breath-holding, and 57 s of self-paced breathing (see Figure 1). This protocol was measured three times, with a 30 min interval between the second and third repetitions, during which the participants were removed from the scanner. The breathing instructions were presented visually via a wall-mounted display and a mirror affixed to the patient’s head coil. The presentation of scanner-triggered stimuli was facilitated using Presentation V20.1 (Neurobehavioral Systems, Berkeley, CA, USA). Respiratory movements were measured using a pneumatic abdominal belt. In instances where respiratory movements were detected during the bh periods, it was inferred that there was no participation. Consequently, participants with absent respiratory recordings were excluded from the analysis.

### 2.3. fMRI Data Processing

The images were subjected to processing using Statistical Parameter Mapping (SPM12) (The Wellcome Department of Imaging Neuroscience, London; https://www.fil.ion.ucl.ac.uk/spm/ accessed on 19 September 2024) running on MATLAB (R2018b (The MathWorks, Inc., Natick, MA, USA; http://www.mathworks.com accessed on 19 September 2024). Initially, the DICOM (Digital Imaging and Communications in Medicine) images were converted into the analyze format in NIfTI (Neuroimaging Informatics Technology Initiative) and subsequently slice-time-corrected to compensate for the varying acquisition times for the images. The images were then realigned to correct for subject head movement and normalized to the standard MNI (Montreal Neurological Institute) space.

**Figure 2:**
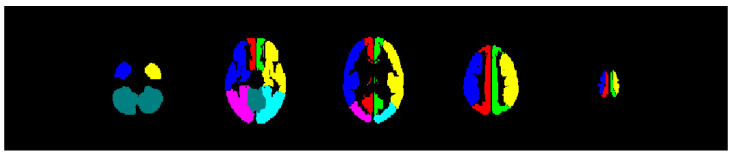
The volumes of interest evaluated based on the vascular territories of the anterior cerebral arteries (right: red; left: green), the middle cerebral arteries (right: blue; left: yellow), the posterior cerebral arteries (right: pink; left: turquoise), and the cerebellum (dark green).

Two different sets of predefined volumes of interest (VOIs) were used for further data evaluation. One set of VOIs included 120 anatomical brain volumes (VOI_anatomical_). The other set of VOIs involved six vascular territories (the anterior cerebral artery (ACA), the middle cerebral artery (MCA), and the posterior cerebral artery (PCA)) based on the arterial transit time flow territories for each brain hemisphere [36,37] and the cerebellum (VOI_vascular_) (see Figure 2). The VOI_regional_ approach was selected for the purpose of investigating the consistency and reproducibility of the method, on the basis that the fine structure of these regions would facilitate a detailed analysis of the method. The VOI_vascular_ were utilized as they appeared to be more relevant to the clinical application of bh-fMRI to indicate the revascularization of individual arteries.

All further processing of the data was performed using in-house scripts programmed in MATLAB (R2018b (The MathWorks, Inc., Natick, MA, USA; http://www.mathworks.com accessed on 19 September 2024)). First, the mean cerebellar signal time course was examined for each of the seven bh periods to ensure compliance, as suggested in previous studies on MMA patients [31]. At this stage, periods with an insufficient cerebellar signal peak due to a lack in compliance could be excluded. The signal time course of the included periods was then averaged. Subsequently, the data were detrended to remove linear signal changes over time. Finally, the percentage BOLD signal change relative to the baseline was calculated for each VOI.

**Figure 3:**
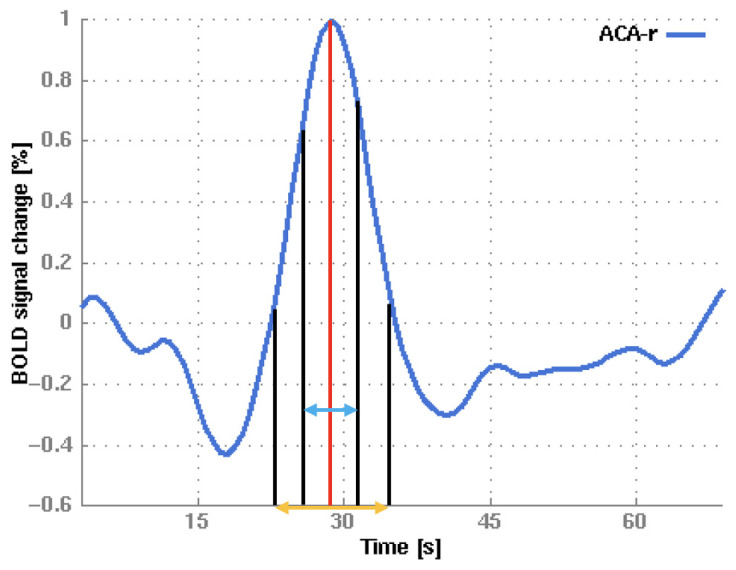
A depiction of the analysis of different intervals.

The primary metric that we focused on was the percentage signal change (PSC). The time point of the cerebellar signal peak was used as a reference, as the time points for the signal peaks in different VOIs differ slightly, and the cerebellum was recently proposed as a reference region for patients with MMA [11]. The PSC in each region at the time point of the cerebellar peak is referred to as the ±0 s interval around the peak (the ±0 s IAP) (see Figure 3). Furthermore, a range of other temporal interval sizes were applied, and the PSC was averaged within a ±3 s interval around the peak (IAP) and a ±6 s IAP (see Figure 3).

A second metric that we used was the time to peak (TTP), which is defined as the time interval from the commencement of the bh period to the occurrence of the maximum PSC in the cerebellum (see Figure 3). However, we primarily focused on the PSC rather than on the TTP as it seemed to have higher clinical importance, and there was more comparable literature [38].

### 2.4. The Statistical Analysis

First, the mean PSC values for the VOI_region_ at all interval sizes were calculated and compared using a Friedman test. If significance was detected, a post hoc analysis with the Wilcoxon test using Bonferroni-corrected *p*-values was conducted.

To assess the inter-session intra-personal reproducibility of the PSC, the ICC of the PSC in the VOI_region_ across different sessions was calculated. We used a two-way random effects model of the ICC (2,k). We compared the ICC of the individual sessions with each other and the ICC between all three sessions. The ICC values were interpreted as follows [39]: poor: <0.4; fair: 0.41–0.59; good: 0.6–0.74; excellent: >0.75. To assess the reproducibility across demographic subgroups, the ICCs in a ±0 s IAP across all three sessions were additionally calculated separately for different sex (male, female) and age groups (≤30, 31–59, ≥60 years).

The influence of different interval sizes on the inter-session intra-personal reproducibility was examined by calculating the ICC separately for each interval size. A Friedman test was performed to determine whether there were significant differences between the methods. If significance was detected, a post hoc analysis with a Wilcoxon test and Bonferroni correction was conducted to identify which methods differed significantly.

Next, to assess intra- and interpersonal variability in the PSC and the TTP values in VOI_vascular_, the coefficient of variation (CV) was evaluated following the established formula [40]:

(1) $$CV=\frac{\sigma }{\mu }\times 100\left(\%\right)$$

where μ is the mean, and σ is the standard deviation (SD).

We calculated the intra-personal CV (CV_intra_) and the inter-personal (CV_inter_). The CV_intra_ represents the variability between different sessions for each individual subject where low variability is expected, accounting for potential measurement errors. The CV_inter_ signifies inter-personal differences within the study population and may help to detect random or systematical errors. A threshold of 33% was considered the maximum acceptable value for normally distributed values [35,41].

To investigate the influence of different interval sizes, the CV_intra_ and CV_inter_ were calculated separately for each interval size. A Friedman test was performed to test for significant differences between the methods, and a post hoc analysis with a Wilcoxon test and Bonferroni-corrected *p*-values was conducted.

The effect size values r were interpreted as follows: r > 0.1 = small effect; r > 0.3 = medium effect; r > 0.5 = large effect [42].

A *p*-value of less than 0.05 was considered to be statistically significant for each primary hypothesis test.

## 3. Results

The general patient data can be found in Table 1. In this study, 49 out of 50 healthy participants successfully completed the breathing intervention with minimal difficulty encountered. One older participant (aged > 70 years) did not perform the task properly, as seen through the belt measurement, and was excluded from the analysis. Of the included participants, 5.3% of the breath-hold periods were excluded after examination of the signal time courses because of an insufficient increase in the BOLD signal response of the cerebellum, indicating insufficient patient cooperation during this specific period. The most frequently reported issue was that the participants often experienced disruption to their natural breathing rhythm, specifically during the exhale phase. When the “exhale” command coincided with natural exhalation in their breathing cycle, the subsequent bh period was perceived as slightly more challenging.

### 3.1. The BOLD Signal Course

**Figure 4:**
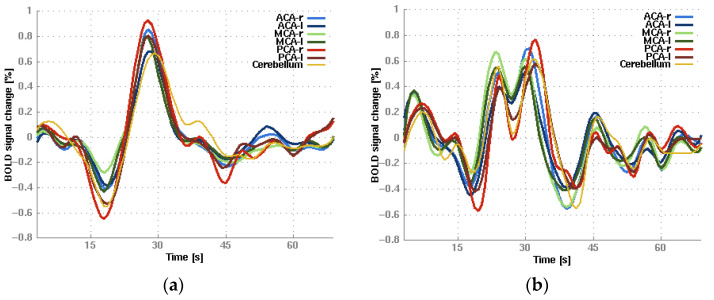
Exemplary bh-fMRI BOLD signal time courses for each VOI with (**a**) one signal peak and (**b**) two partially overlapping signal peaks. ACA-r: right anterior cerebral artery; ACA-l: left anterior cerebral artery; MCA-r: right middle cerebral artery; MCA-l: left middle cerebral artery; PCA-r: right posterior cerebral artery; PCA-l: left posterior cerebral artery.

Exemplary BOLD signal time courses are shown in Figure 4. For most of the BOLD signal time courses, only one single peak was observed (see Figure 4a). In some cases, the BOLD signal courses showed two consecutive signal peaks occurring in close temporal proximity, partially overlapping (see Figure 4b).

**Figure 5:**
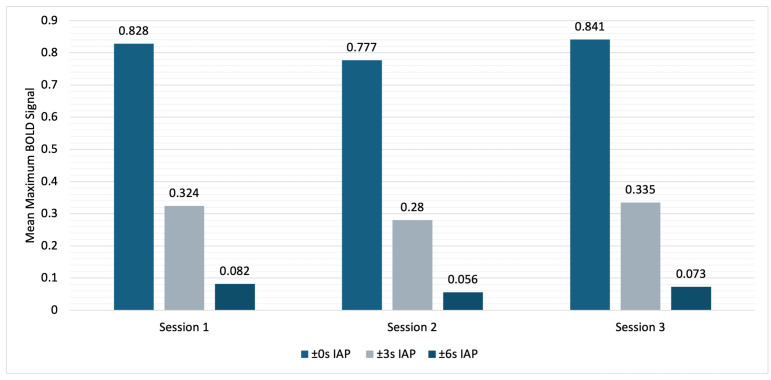
Comparison of the mean PSCs using different time interval sizes.

As illustrated in Figure 5, there were distinct and significant differences in the PSCs when using different time intervals (X^2^ = 6.000, df = 2, *p* = 0.05, r = 0.35). The mean PSC in a ±0 s IAP (0.815% ± 0.258%) was significantly higher than the mean PSC with a ±6 s IAP (0.070% ± 0.046%) (*p* = 0.043, corrected). The comparison between the mean PSCs in the ±0 s IAP and the ±3 s IAP (0.313% ± 0.121%) was not significant (*p* = 0.662, corrected), nor was the comparison between the ±3 s IAP and the ±6 s IAP (*p* = 0.662, corrected).

### 3.2. The Inter-Session Intra-Personal Reproducibility of the Changes in the BOLD Signals 

Table 2 presents the ICCs of the PSCs between all sessions and across all intervals. Independent of the sessions and the interval size, all of the ICC values exceeded the 0.75 threshold, indicating excellent intra-day reproducibility according to Cicchetti et al. [43]. The ICC value using the ±0 s IAP was 0.887 when comparing all three sessions. The ICC between the first and second session was a little higher (0.849) compared to the ICC values for session 1 and 3 (0.839) and session 2 and 3 (0.832), which included a break and patient repositioning in between.

**Figure 6:**
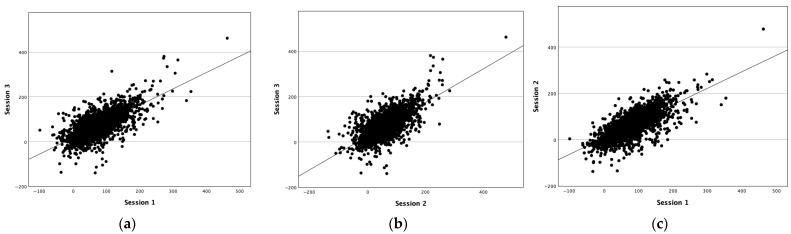
A scatter plot illustrating the correlation of the PSC in the VOI_region_ between the sessions. Each dot represents the PSC in % in one of the VOI_region_ in one session using the ±0 s IAP. (**a**) A scatter plot result for session 1 and 3. (**b**) A scatter plot for session 2 and 3. (**c**) A scatter plot for session 1 and 2.

The intra-personal correlation in the PSC when using the ±0 s IAP across different sessions is illustrated through the scatter plots in Figure 6.

**Figure 7:**
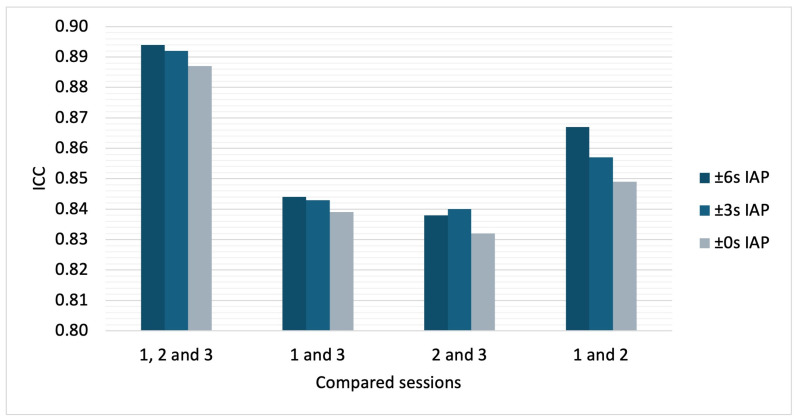
A comparison of the intraclass correlation coefficient (ICC) of the bh-fMRI BOLD signal change between different sessions and across different interval sizes; IAP = interval around the peak.

The ICC of the PSC differed significantly depending on the size of the temporal interval (X^2^ = 6.500; df = 2, *p* = 0.039, r = 0.36), whereby the ICC values decreased slightly as the time interval was shortened (see Figure 7). The post hoc analysis revealed significant differences between the ICC for the ±6 s IAP and that for the ±0 s IAP (*p* = 0.04, corrected) but not between the ICC for the ±6 s IAP and that for the ±3 s IAP (*p* = 1.00, corrected) or between the ICC for the ±3 s IAP and that for the ±0 s IAP (*p* = 0.231, corrected). The ICC values in a ±0 s IAP across all age and sex subgroups ranged from 0.838 to 0.919, indicating excellent reproducibility (Table A1).

### 3.3. Inter-Session Intra-Personal Variability and Intra-Session Inter-Personal Variability

The CV_intra_ of both the ±0 s IAP and the TTP was lower than the CV_inter_ (see Table 3). The mean CV_intra_ in a ±0 s IAP was 14.52% ± 8.54% for the PSC in a ±0 s IAP and 4.84% ± 3.94% for the TTP (see Table 3). In the ±0 s IAP, the mean CV_inter_ remained below the fiducial threshold of 33% at 32.0% ± 2.33%, indicating an acceptable level of variability [35,41]. For the TTP, the CV_inter_ remained consistently below 10% in each session, with a mean of 9.75% ± 2.76% (see Table 3).

A longer time interval resulted in an increase in the CV_intra_ and CV_inter_. The CV_intra_ values remain below the 33% threshold but still increase up to 21.17% ±25.48% for the ±3 s IAP and 18.94% ± 97.97% for the ±6 s IAP. In the case of the ±3 s IAP and the ±6 s IAP, the CV_inter_ surpasses the upper limit of 33%. Comparing the CV_inter_ of the ±0 s IAP to that for the ±3 s IAP, it increases by 21.88%, and comparing the PSC in the ±0 s IAP to that for the ±6 s IAP, the mean variability increases by 109.38% (see Table 3).

## 4. Discussion

### 4.1. The Reproducibility of and Variability in the Increase in the BOLD Signal After Short Bh Periods

Assessing the reproducibility of bh-fMRI is essential to ensure the reliability and validity of CVR measurements across different sessions and populations. CVR mapping is increasingly used in both research and clinical settings to assess cerebrovascular health and identify early dysfunction in conditions such as stroke [6,7,44], MMA [10,11,45,46], and extracranial artery stenosis [8,9,47]. CVR measurements hold promise for perioperative risk stratification, treatment monitoring, and the prediction of cognitive outcomes [48,49]. Beyond these established applications, CVR mapping is also increasingly used in hypertension [12], traumatic brain injury [13,14], epilepsy [50], and even neurooncological imaging [51,52].

In light of this growing clinical relevance, a robust evaluation of reproducibility is critical for future clinical and research applications of bh-fMRI. Our study contributes to this ongoing effort by systematically evaluating the reproducibility of key CVR parameters using a short, tolerable breath-hold paradigm. In doing so, we respond to the increasing demand for protocols that balance signal robustness with patient compliance and translational applicability.

Therefore, in this study, we focused on the intra-personal inter-session reproducibility and performed three bh-fMRI scans with a 30 min interval between the second and third repetitions, during which time the participants were removed from the scanner. Our results extent the findings by Magon et al. (2009) [35], who reported acceptable reproducibility (CV_intra_) for a 9 s end-inspiratory breath-hold paradigm. In contrast to Magon et al., we used end-expiratory breath-holds, which are physiologically more stable and less prone to BOLD signal variability compared to end-inspiratory holds [53,54]. As a further improvement, we report not only the CV_intra_ but also the ICC values—a standard for reproducibility assessments [35]. Additionally, we evaluated the PSC at different time intervals. By reporting both the CV_intra_ and ICC for relevant parameters in a clinically easily feasible end-expiratory breath-hold protocol, we provide a more comprehensive and application-oriented evaluation of protocol stability.

Our results revealed excellent intra-day reproducibility according to Cicchetti et al., with ICC values distinctly higher than 0.75 [39]. These results indicated not only excellent reproducibility across all sessions but also between individual sessions. When the first and second sessions were compared with the third, the ICC values were slightly lower than the ICC values between the first and second sessions. As sessions 1 and 2 were directly completed one after the other, it is not surprising that the reproducibility tended to be higher. However, even if a break was included between the sessions and the participants left the scanner, the ICC values were also high and did not differ significantly from the values without a break. High ICC values were measured regardless of age and sex.

Comparable ICC values were obtained in similarly conceptualized studies [34,55]. Studies by Dlamini et al. achieved ICC values in the range of good to excellent reproducibility [55], while Peng et al. only documented superiority compared to the acceptable limit [34]. A possible explanation for the higher reproducibility of our measurement methodology compared to that in Dlamini et al. is that their study was conducted in children with MMA, with two participants also suffering from sickle cell anemia, which can influence BOLD signals [55]. Since only a few studies have investigated the reproducibility of bh-fMRIs to date, a comparison with CO_2_-triggered studies is appropriate. The intra-day reproducibility values for these methods also showed excellent ICC values, such as in studies by Kassner et al. (ICC = 0.92 in the gray matter (GM); ICC = 0.88 in the white matter (WM)) [56] and Leung et al. (ICC = 0.857 in the GM; ICC = 0.719 in the WM) [57]. Good inter-day reproducibility values were achieved for the WM (ICC = 0.66 [56]; ICC = 0.719 [57]), while the values in the GM remained excellent (ICC = 0.81 [56]; ICC = 0.776 [57]). It is known that reproducibility is lower in the WM [34,55,56,58,59], as regions close to the ventricular system are prone to signal loss and distortion due to susceptibility artefacts. Our results are within the same range as the fMRI studies using CO_2_ inhalation mentioned above. Bh-fMRI has the advantage that it does not require specialized equipment and is not associated with increased discomfort, while it gives comparable results to [^15^O]water PET measurements [11,31]. Therefore, it seems to be a good alternative to CO_2_-triggered fMRI.

The CV_intra_ was calculated because it indicates how stable the measurements are within a person, i.e., whether the results vary due to measurement errors or other random fluctuations or whether they are consistent. Therefore, it can also be seen as a tool for evaluating intra-personal reproducibility. The mean CV_intra_ for the PSC using a ±0 s IAP and the TTP was distinctly less than the threshold of 33% defined by Johnson and Welch [41], which indicates acceptable variability in the BOLD response and its timing across all sessions.

In this study, the CV_inter_ was mainly used as a tool to depict the variability in the study population and to detect systematical or random measurement mistakes. The mean CV_inter_ was below the maximum acceptable value of 33%, indicating that the technique seems to be reliable and robust regarding mistakes. A lower CV_intra_ compared to CV_inter_ has already been observed in previous studies [32,34,35,60,61]. This difference is an expected phenomenon because the CV_inter_ is influenced by the physiological differences between subjects. The present study comprised a heterogeneous study population with a balanced gender ratio and a wide age range. Age- and gender-specific differences in CVR have already been found in previous studies, which could explain the higher CV_inter_ values [56,62,63,64]. Self-controlled breathing affects reproducibility [65], as the BOLD signal is highly dependent on the depth of inspiration [54], and this could have led to heterogeneous perturbations in the BOLD signals during the relatively short breathing pauses. Nevertheless, it is essential to emphasize that the CV_inter_ values remained below the acceptable threshold. The aforementioned arguments seek to elucidate tendencies in the values which exert no direct influence on the evaluation of reproducibility.

Our results are consistent with those from comparable studies [55]. Peng et al. also determined CV_intra_ and CV_inter_ values below 33% using bh periods of 20 s [34]. In contrast, another study by Magon et al. found that only the CV_intra_ for the TTP and the PSC was below this limit, also using bh periods of 9 s [34]. Other parameters such as the area under the curve exceeded this value [34]. This indicates that reproducibility might not be the same for all measurement parameters.

To conclude, it is important to mention that the bh-fMRI technique can be conducted in several different ways, and variations in the breath-hold paradigm and the technical setting may have an influence on reproducibility. Regarding the design of the breath-hold paradigms, especially the performance in end-inspiration vs. end-expiration, the use of paced breathing, the duration of the bh period, and the scan length [59], as well as repetition [66] of the measurements and the task while holding one’s breath [59], have an impact. As concerns the technical setting, the field strength [34] and the use of a regressor, which includes the integration of end-tidal CO_2_ (EtCO_2_) as a regressor [2], is discussed as having an impact on reproducibility. Normalization with CO_2_ may eliminate physiological fluctuations such as fluctuations in respiratory rate and lung function and may contribute to standardization and inter-study comparability [20,67]. Nevertheless, some other studies have reported that end-tidal CO_2_ correction may, in certain cases, lead to a decrease in reproducibility [66,68]. Furthermore, this approach requires additional measurement devices, which did not align with our study’s objective of developing a clinically feasible breath-hold paradigm. In conclusion, our study’s bh-fMRI design yielded excellent reproducible outcomes. The ICC and CV_intra_ are crucial instruments for the assessment of reproducibility, and both indicators demonstrate a high degree of reliability regarding our bh-fMRI technique. The novel insights derived from this study may contribute to the refined conceptualization of a breathing paradigm, taking into account the aforementioned technical influences.

### 4.2. The Influence of the Temporal Interval Size on Reproducibility and Variability

Another aim of this study was to investigate the influence of the temporal interval size on reproducibility and variability. Our hypothesis was that using a larger time interval, individual differences in the complex BOLD signal curve could be compensated for, leading to more reproducible results. Our study revealed significantly higher ICC values for smaller time intervals with a medium effect size, while all values indicated an excellent degree of reproducibility according to Cicchetti et al. [39]. However, the PSC was significantly higher using the ±0 s IAP than the PSC using a longer time interval, and the CV_inter_ and CV_intra_ increased with a longer time interval. At first glance, an increase in the CV_intra_ may appear paradoxical. However, a thorough examination of the data reveals that negative BOLD signal values are more prevalent for the ±3 s and ±6 s IAPs (see Figure 3). The aforementioned factors result in a reduction in the mean and an increase in the CV_intra_. Consequently, longer time intervals do not seem to be advisable for future clinical use.

### 4.3. Limitations

Certain limitations must be considered when interpreting the results. The objective of this study was to implement a feasible and simple application; however, it would not have been possible to achieve this at the desired level if an attempt had been made to measure CO_2_ as a regressor. It can be posited that the absence of appropriate calibration may have been a contributing factor to the relatively high CV_inter_ values observed. Furthermore, the use of a relatively short breath-hold duration may have limited the strength of the hypercapnic stimulus. While longer durations can enhance BOLD signal amplitudes [35,53], they are also associated with decreased compliance and increased motion artifacts. Our decision to use a brief end-expiratory protocol reflects a conscious trade-off aiming to optimize the clinical feasibility and standardization, even if it may come at the expense of absolute signal strength.

Another limitation is that this study was conducted using a fixed 9 s breath-hold duration without direct comparisons with shorter or longer breath-hold protocols. While this decision was based on its intended clinical feasibility, future research should address the reproducibility of varying the breath-hold lengths to optimize the CVR paradigms further.

The quality of the collected data is contingent on participant compliance and requires active cooperation [1,11,31,69]. Individual differences in the execution of the breath-holding maneuver may influence the BOLD signal response. Although compliance can be objectively verified using a breathing belt, this method does not capture potential variations in internal muscle activity and CO_2_ content, changes in intrathoracic pressure, or variations in breath depth and length, which could affect aspects of the BOLD response [22,66,70,71]. Because this technique can be influenced by the aforementioned factors, we observed cases in which it was rather challenging to detect the cerebellar peak, as a multiphasic signal course pattern was shown.

Despite the attempt to encompass a broad age range, this study did not include children. Moreover, caffeine consumption [72] and daytime-dependent fluctuations in CVR were not regarded [73], and our visual paradigm may be attributable to individual differences in the coverage of the visual field or physiological fluctuations [74].

Finally, it should be noted that from our analysis thus far, only statements about the intra-day reproducibility can be made, which is often greater than the inter-day reproducibility. Future studies should focus on investigating this issue.

## 5. Conclusions

Our results provide strong evidence for the high intra-day reproducibility of breath-hold-induced BOLD signal changes using short bh periods, as demonstrated by high ICC values and low CV_intra_ values, even after patient repositioning. This supports the robustness of this method for evaluating cerebrovascular function in both research and clinical settings. Future studies should focus on the between-day reproducibility.

## Figures and Tables

**Table 1:** General characteristics of study population.

**Participants, n**	49 *
**Included bh-fMRI datasets**	147
**M:F ratio**	1.04:1
**Mean age (range)**	45.5 (20–74)

* Data after the exclusion of a participant due to insufficient compliance.

**Table 2:** ICCs and 95% confidential intervals across different interval sizes.

Session	±6 s IAP	±3 s IAP	±0 s IAP
**1, 2 and 3**	0.894 (0.890–0.899)	0.892 (0.887–0.897)	0.887 (0.882–0.892)
**1 and 3**	0.844 (0.836–0.852)	0.843 (0.834–0.851)	0.839 (0.830–0.847)
**2 and 3**	0.838 (0.829–0.846)	0.840 (0.832–0.848)	0.832 (0.823–0.840)
**1 and 2**	0.867 (0.859–0.873)	0.857 (0.849–0.864)	0.849 (0.840–0.856)

**Table 3:** The intra-personal and inter-personal variation in the ±0 s IAP and the TTP and its standard deviation.

	PSC	TTP
	**CV_intra_**	
±0 s IAP	14.52% ± 8.54%	4.84% ± 3.94%
±3 s IAP	21.17% ± 25.48%	-
±6 s IAP	18.94% ± 97.97%	-
	**CV_inter_**	
±0 s IAP	32.0% ± 2.33%	9.75% ± 2.76%
±3 s IAP	39.0% ± 4.51%	-
±6 s IAP	67.0% ± 13.25%	-

CV_intra_ = intra-personal coefficient of variation; CV_inter_ = inter-personal coefficient of variation.

## Data Availability

In order to safeguard the confidentiality of the participants, the data pertaining to this study are currently withheld from public access. The data can be shared upon request.

## Abbreviations

The following abbreviations are used in this manuscript:

ACA	A. cerebri anterior
bh-fMRI	Breath-hold functional MRI
bh periods	Breath-hold periods
BOLD fMRI	Blood-oxygenation-level-dependent functional magnetic resonance imaging
CBF	Cerebral blood flow
CMRO_2_	Cerebral metabolic rate of oxygen
CVR	Cerebrovascular reactivity
CV	Coefficient of variation
IAP	Interval around the cerebellar peak
ICC	Intraclass correlation coefficient
MCA	A. cerebri media
MMA	Moyamoya Angiopathy
PCA	A. cerebri posterior
PET	[^15^O]water positron emission tomography
PSC	Percentage signal change
TTP	Time to peak
VOI	Volume of interest

## Appendix A

**Table A1:** ICCs and 95% confidential intervals across all three measurements in a ±0 s IAP for sex and age.

Age	Sex	
	Male	Female
≤30	0.863 (0.842–0.881)	0.919 (0.911–0.927)
31–59	0.841 (0.824–0.856)	0.887 (0.875–0.899)
≥60	0.903 (0.893–0.912)	0.838 (0.811–0.862)
